# Potential of Neural Stem Cells for the Treatment of Brain Tumors

**DOI:** 10.4137/cmo.s747

**Published:** 2008-06-09

**Authors:** P. Taupin

**Affiliations:** 1 Fighting Blindness Vision Research Institute; 2 Dublin City University

**Keywords:** cancer, cellular therapy, nervous system, gene therapy

## Abstract

Neural stem cells (NSCs) are self-renewing multipotent cells that generate the main phenotypes of the nervous system, neurons, astrocytes and oligodendrocytes. As such they hold the promise to treat a broad range of neurological diseases and injuries. Neural progenitor and stem cells have been isolated and characterized in vitro, from adult, fetal and post-mortem tissues, providing sources of material for cellular therapy. However, NSCs are still elusive cells and remain to be unequivocally identified and characterized, limiting their potential use for therapy. Neural progenitor and stem cells, isolated and cultured in vitro, can be genetically modified and when transplanted migrate to tumor sites in the brain. These intrinsic properties of neural progenitor and stem cells provide tremendous potential to bolster the translation of NSC research to therapy. It is proposed to combine gene therapy and cellular therapy to treat brain cancers. Hence, neural progenitor and stem cells provide new opportunities for the treatment of brain cancers.

## Introduction

Cancers result from genetic and epigenetic changes in normal growth-controlled cells; the cells become unable to terminally differentiate, to control their ability to proliferate, and acquire the ability to invade other tissues and spread through the body or metastasize (Cahill et al. 1999). In the central nervous system (CNS), tumors that originate in the brain or spinal cord are called primary tumors. Primary brain tumors are named according to the type of cells or the part of the brain in which they begin. Most primary tumors originate from out-of-control growth among cells that surround and support neurons. The most common primary brain tumors are gliomas. They begin in glial cells. There are many types of gliomas, like astrocytoma in which the tumor arises from a population of glial cells called astrocytes, ependymoma in which the tumor arises from cells that line the ventricles or the central canal of the spinal cord, oligodendroglioma in which the tumor arises from oligodendrocytes. Some types of brain tumors do not begin in glial cells, like medulloblastoma that usually arises in the cerebellum, meningioma that arises in the meninges. When cancer cells spread to the brain from another organ, like the lung or breast, the tumors in the brain are called secondary tumors or metastatic tumors. Secondary tumors in the brain are more common than primary brain tumors. Brain tumors are rated by grade, from low grade (grade I) to high grade (grade IV), corresponding to the way the cells look under a microscope. Cells from high-grade tumors look more abnormal, and generally grow faster than cells from low-grade tumors. The CNS is housed within rigid, bony quarters (i.e. the skull and spinal column), so any abnormal growth, whether benign or malignant, can place pressure on sensitive tissues, impair function and be life threatening. Surgery, radiation and chemotherapy are the three most commonly used treatments for brain tumors (Kaal et al. 2005).

## Neural Stem Cells and Cellular Therapy

Stem cells are the building blocks of the body (Potten and Loeffler, 1990). Pluripotent stem cells can generate cells participating to the three germ layers of the individuals, the ectoderm, mesoderm and endoderm, and the germ cells. Multipotent stem cells generate lineage specific cell types restricted to the tissues from which they are derived. Pluripotent stem cells are present in embryonic tissues and multipotent stem cells are present in fetal and adult tissues. During development, multipotent stem cells contribute to the formation of the tissues and in the adult they contribute to homeostasis of the tissues and regeneration after injury. In the mammalian nervous system, during development, newborn neuronal cells originate from NSCs in the ventricular zone (Angevine, 1965). In the adult brain, contrary to a long-held dogma, neurogenesis occurs in the adult brain and NSCs reside in the adult CNS (Gross, 2000), in various species including humans (Taupin and Gage, 2002).

Neural progenitor and stem cells have been isolated and characterized in vitro from various regions of the fetal, adult and post-mortem brain tissues, from various species including humans (Ryder et al. 1990; Reynolds and Weiss, 2002; Gage et al. 1995; Roy et al. 2000; Palmer et al. 2001). Neural progenitor cells are multipotent cells with limited proliferative capacity. Because of their potential to generate the main phenotypes of the nervous system, NSCs have the potential to treat a broad range of diseases and injuries of the nervous system, like neurodegenerative diseases, strokes and spinal cord injuries (Taupin et al. 2006).

## Potential of Neural Progenitor and Stem Cells for Brain Tumor Therapy

Neural progenitor and stem cells have intrinsic properties that make them particularly interesting and valuable for therapy (Dwain et al. 2006). Neural progenitor and stem cells isolated from fetal tissues and expanded in vitro migrate to degenerated, injured and tumor sites in the nervous system, when transplanted in the CNS, or when administered by systemic injection, in blood vessels or in the cerebrospinal fluid, by injecting cells into the 4th ventricle in the rat (Aboody et al. 2000; Brown et al. 2003; Macklis, 1993; Wu et al. 2002; Fujiwara et al. 2004; Jeon et al. 2008). A recent study has reported that systemic injection of neural progenitors and stem cells may provide significant clinical benefit in an animal model of multiple sclerosis (Pluchino et al. 2003). These properties considerably broadened the spectrum of diseases and injuries that can be treated using neural progenitor and stem cells, like Alzheimer’s disease, Huntington’s disease and multiple sclerosis—where the degeneration is widespread and for which “classic” cell transplantation may not be suitable.

Such properties of NSCs can then be used not only for the treatment of neurodegenerative diseases where the degeneration is widespread, but also as mode of delivering NSCs for cellular therapy, avoiding the practice of surgical procedures, and their associated risks and secondary effects. Systemic injection and injection through the cerebrospinal fluid are regarded as a promising ways to administer NSCs for cellular therapy, particularly for the treatment of spinal cord injury, with minimum damages to the host tissue and limiting the surgical procedure (Bai et al. 2003; Bakshi et al. 2004).

Neural progenitor and stem cells isolated from fetal and adult tissues and expanded in vitro can be genetically engineered (Gage et al. 1995; Liu et al. 1999). Niemann-Pick’s disease is a lysosomal storage disorder in which deficiency of acid sphingomyelinase leads to the intracellular accumulation of sphingomyelin and cholesterol in lysosomes (Kolodny, 2000). Genetically engineered neural progenitor and stem cells expressing acid sphingomyelinase have been reported to reverse lysosomal storage pathology in animal models of Niemann-Pick’s disease (Shihabuddin et al. 2004), confirming the potential of NSCs to serve as a gene transfer vehicle for the treatment of CNS pathology, particularly for lysosomal storage diseases. Hence, genetically engineered neural progenitor and stem cells extend their potential use for the treatment of neurological diseases caused by a genetic deficiency, but also to promote neuronal survival in neurodegenerative diseases (Chen et al. 2007).

The properties of neural progenitor and stem cells to be genetically engineered and to migrate to tumor sites have been proposed for the treatment of brain tumors. It is proposed to genetically engineer neural progenitor and stem cells with “suicide genes”, like genes coding for cytolytic activities or anti-tumor cytokines. Transplanted or peripherally administered, such genetically engineer neural progenitor and stem cells would then migrate to tumor sites where they would attack and destroy tumor cells (Yip et al. 2003; Shah et al. 2005).

## Perspectives for the Treatment of Brain Tumors

The cause of most primary tumors remains unknown, though in some cases, specific genetic diseases (e.g. neurofibromatosis), exposure to radiation or cancer-causing chemicals are suspected. Because of their properties to live for long period of time and divide over time (self-renewal), mutations and epigenetic changes would accumulate in stem cells, leading to aberrant growth and tumor formation (Kondo, 1983).

It is proposed that the carcinogenic process may start in a stem cell (Trosko and Chang, 1989; Lapidot et al. 1994). According to this theory, tumors would originate from the transformation of normal stem cells to cancer cells, i.e. cancer stem cells (CSCs) (Reya et al. 2001). Recently, CSCs have been isolated and characterized prospectively from brain tumors (Singh et al. 2004a). The identification of CSCs from brain tumors or brain tumor stem cells (BTSCs) has been made by applying the principles of stem cell biology to brain tumor cell populations (Hemmati et al. 2003; Pardal et al. 2003; Singh et al. 2003; Vescovi et al. 2006; Kong et al. 2008). Though there are accumulated evidences for a stem origin for cancers, the hypothesis about NSCs or BTSCs as a cell population highly susceptible for neoplastic transformation, and responsible for tumor recurrence after local treatments in the CNS, is still highly controversial and remains to be further confirmed (Singh et al. 2004b; Uchida et al. 2004; Jackson and Alvarez-Buylla, 2008). The stem cell theory of carcinogenesis predicts that cancer cells and normal stem cells may share common mechanisms and pathways (Jandial et al. 2008). Therefore, the identification of BTSCs may also lead to a better understanding of the mechanisms leading to carcinogenesis and therapy (Al-Hajj et al. 2004; Ruiz-Lozano and Rajan, 2007). However, though the evidences are compelling, cancers and brain cancers may not be a disease originating purely in stem cells. The origin of brain tumors remains therefore to be further investigated and confirmed.

## Conclusion

The confirmation that adult neurogenesis occurs in the adult brain and NSCs reside in the adult CNS suggests that the adult brain may be amenable to repair. Despite intense work, NSCs are still elusive cells and remain to be fully identified and characterized in vitro and in vivo. Further identification and characterization of NSCs are prerequisite to bring NSC research to therapy. Properties of NSCs to migrate to tumors and to be genetically engineered provide a promising opportunity to treat brain tumors. Neural progenitor and stem cells have tremendous consequences not only for our understanding of brain development and cellular therapy, but also for the origin of brain tumors. The isolation and characterization of BTSCs suggest that brain tumors may be stem cell diseases, which may lead to the development of new strategies to cure brain tumors. Several issues would need to be addressed before stem cells could be employed for the treatment of brain tumors. Among them, what are the secondary risks of using stem cells as vector to attack and destroy tumor cells? What strategies can be devised to specifically target BTSCs?
